# Assessment of the Severity of COVID-19 on the Basis of Examination and Laboratory Diagnostics in Relation to Computed Tomography Imagery of Patients Hospitalised Due to COVID-19—Single-Centre Study

**DOI:** 10.3390/healthcare12141436

**Published:** 2024-07-18

**Authors:** Tomasz Ilczak, Szymon Skoczynski, Ewa Oclon, Mirosław Kucharski, Tomasz Strejczyk, Marta Jagosz, Antonina Jedynak, Michał Wita, Michał Ćwiertnia, Marek Jędrzejek, Mieczysław Dutka, Wioletta Waksmańska, Rafał Bobiński, Roch Pakuła, Marek Kawecki, Paweł Kukla, Szymon Białka

**Affiliations:** 1 Department of Emergency Medicine, Faculty of Health Sciences, University of Bielsko-Biala, 43-309 Bielsko-Biała, Poland; mcwietnia@ubb.edu.pl (M.Ć.); mkawecki@ubb.edu.pl (M.K.); 2 Department of Lung Diseases and Tuberculosis, Faculty of Medical Sciences in Zabrze, Medical University of Silesia, 40-752 Katowice, Poland; simon.mds@poczta.fm; 3 Centre for Experimental and Innovative Medicine, Laboratory of Recombinant Proteins Production, University of Agriculture in Krakow, 30-059 Kraków, Poland; 4 Department of Animal Physiology and Endocrinology, University of Agriculture in Krakow, Al Mickiewicza 24/28, 30-059 Krakow, Poland; miroslaw.kucharski@urk.edu.pl; 5 Leszek Giec Upper-Silesian Medical Centre, Medical University of Silesia in Katowice, 40-287 Katowice, Poland; strejson@poczta.onet.pl; 6 Students’ Scientific Association, Department of Anaesthesiology and Intensive Care, Faculty of Medical Sciences in Zabrze, Medical University of Silesia, 40-287 Katowice, Poland; marta.malgorzata.jagosz@gmail.com; 7 Students’ Scientific Association, Department of Pneumonology, School of Medicine in Katowice, Medical University of Silesia, 40-287 Katowice, Poland; s77596@365.sum.edu.pl; 8 Department of Cardiology, Faculty of Medical Sciences in Katowice, Medical University of Silesia, 40-287 Katowice, Poland; michalwita21@gmail.com; 9 Division of Cardiology and Structural Heart Diseases, Medical University of Silesia, 40-287 Katowice, Poland; jedrzejekmarek@gmail.com; 10 Department of Biochemistry and Molecular Biology, Faculty of Health Sciences, University of Bielsko-Biala, 43-309 Bielsko-Biała, Poland; mdutka@ath.bielsko.pl (M.D.); rbobinski@ubb.edu.pl (R.B.); 11 Department of Public Health, Faculty of Health Sciences, University of Bielsko-Biala, 43-309 Bielsko-Biała, Poland; wwaksmanska@ubb.edu.pl; 12 Department of Cardiac Surgery, Cardiac and Lung Transplantation, Mechanical Circulatory Support, Silesian Centre for Heart Diseases, 41-800 Zabrze, Poland; 13 Medical College, Jagiellonian University, 31-001 Kraków, Poland; pawel.kukla@uj.edu.pl; 14 Department of Anesthesia and Intensive Therapy, Faculty of Medical Sciences in Zabrze, Medical University of Silesia, 40-287 Katowice, Poland; szymon.bialka@gmail.com

**Keywords:** emergency medical services, medical professionals, emergency procedures, COVID-19 pandemic, computed tomography, laboratory diagnostics

## Abstract

From the moment the SARS-CoV-2 virus was identified in December 2019, the COVID-19 disease spread around the world, causing an increase in hospitalisations and deaths. From the beginning of the pandemic, scientists tried to determine the major cause that led to patient deaths. In this paper, the background to creating a research model was diagnostic problems related to early assessment of the degree of damage to the lungs in patients with COVID-19. The study group comprised patients hospitalised in one of the temporary COVID hospitals. Patients admitted to the hospital had confirmed infection with SARS-CoV-2. At the moment of admittance, arterial blood was taken and the relevant parameters noted. The results of physical examinations, the use of oxygen therapy and later test results were compared with the condition of the patients in later computed tomography images and descriptions. The point of reference for determining the severity of the patient’s condition in the computer imagery was set for a mild condition as consisting of a percentage of total lung parenchyma surface area affected no greater than 30%, an average condition of between 30% and 70%, and a severe condition as greater than 70% of the lung parenchyma surface area affected. Patients in a mild clinical condition most frequently had mild lung damage on the CT image, similarly to patients in an average clinical condition. Patients in a serious clinical condition most often had average levels of damage on the CT image. On the basis of the collected data, it can be said that at the moment of admittance, BNP, PE and HCO_3_^−^ levels, selected due to the form of lung damage, on computed tomography differed from one another in a statistically significant manner (*p* < 0.05). Patients can qualify for an appropriate group according to the severity of COVID-19 on the basis of a physical examination and applied oxygen therapy. Patients can qualify for an appropriate group according to the severity of COVID-19 on the basis of BNP, HCO_3_ and BE parameters obtained from arterial blood.

## 1. Introduction

From the moment the SARS-CoV-2 virus was identified in December 2019, the COVID-19 disease spread around the world, causing an increase in hospitalisations and deaths [1]. From the beginning of the pandemic, scientists tried to determine the major cause that led to patient deaths. Various theories appeared, such as a cytokine storm causing multiple organ failure, thromboembolic disease, sepsis and complications leading to cardiovascular failure [2]. However, in the majority of described cases, death due to COVID-19 has been connected with severe respiratory failure, which depends on the surface area of the lung parenchyma affected by the disease, the intensification of consolidation and micro-embolic complications resulting in a decrease in the respiratory index [3,4]. During increased activity of the SARS-CoV-2 virus, healthcare systems around the world were unable to cope, resulting in extremely long waiting times for the diagnosis and treatment of patients suffering from COVID-19 [5]. There was a lack of staff, combined with their overtiredness, working in epidemiological conditions, as well as extremely limited logistical possibilities for hospitals. One very serious problem was the quick diagnosis of patients in order to determine the severity of damage to the lungs, and qualifying patients for appropriate oxygen therapy and further treatment. From the perspective of speed and precision, the best diagnostic method for assessing the severity of damage to the lungs is computed tomography (CT) [6,7,8]. CT imaging scans are available in the majority of clinics treating COVID-19 patients. What is more, they enable the diagnosis of other potential causes of the worsening of a patient’s condition, such as pulmonary embolism or cancerous growths [9]. It became a problem to find places for patients in the most critical clinical condition requiring immediate treatment or stabilization of basic vital parameters. Conducting bedside CT imaging is severely limited, and transporting seriously ill patients requires a larger number of staff and optimal conditions from an epidemiological point of view for transport logistics, which in times of limited human resources with the unpreparedness of the system became a significant challenge. Unfortunately, in many places, diagnostic imagery using CT had to be delayed at least until the necessary emergency procedures were completed. This resulted in delays in determining the ultimate form of therapy and in determining the prognosis for patients with COVID-19. Image diagnosis is the best and most effective method of assessing lung damage, but unfortunately it is not always available at the early stages of treating COVID-19 patients. For this reason, the background to forming a research model for this paper and for conducting the statistical analysis were diagnostic problems related to difficulties in quickly carrying out CT, and in determining early on the degree of lung damage in COVID-19 patients. A research model was developed to answer the question of whether a patient’s physical examination and basic laboratory diagnostics are sufficient to determine the severity of the disease. The implementation of appropriate oxygen therapy and determination of prognosis for patients with COVID-19 were also evaluated.

## 2. Materials and Methods

### 2.1. Research Hypothesis

The adopted research hypothesis was that the use of a physical examination and basic laboratory diagnosis allowed for the severity of COVID-19 to be assessed upon admittance of a patient to hospital. The verification method for the adopted research hypothesis was assessment of CT, which defined the level of advancement of the disease depending on the percentage of lung parenchyma affected.

### 2.2. Research Model

The research was observatory in nature. The research evaluated the documentation for 95 patients, which included a physical examination, the type of oxygen therapy and breathing assistance used, the results of laboratory tests and the result of imagery diagnosis in the form of CT descriptions. Permission for the research was granted by the ethics committee of the Silesian Medical University (No PCN/CBN/0022/KB1/125/I/21/22, 8 February 2022).

### 2.3. Study Group

The study group comprised patients hospitalised in one of the temporary COVID hospitals. The patients admitted to hospital had a PCR test or antigen test upon admittance confirming infection with SARS-CoV-2. The study group consisted of men and women of various ages and with varying degrees of severity of the disease. The patients required passive or active oxygen therapy or invasive or non-invasive ventilation, which was implemented immediately after or during the physical examination conducted upon admittance. Those in the study group had arterial blood taken upon admittance, and the appropriate parameters were determined. The result of the physical examination, the oxygen therapy used and at a later stage the test results were compared with the patient’s condition in the later CT image and description. To determine the clinical severity of the disease, patients were divided according to the necessity to use passive or active oxygen therapy, or invasive or non-invasive ventilation. Respectively, a mild condition was represented by the use of High-Flow Nasal Oxygen Therapy (HFNO), an average condition by the use of Non-Invasive Ventilation using a face mask (NIV) and a severe condition by the necessity to conduct invasive lung ventilation. Upon admittance of the patient to hospital, CT tomography was carried out on the chest. The result of the test determined the percentage of lung parenchyma damaged by COVID-19, and excluded other complications such as pulmonary embolism or pneumothorax. The point of reference for determining the severity of the patient’s condition in the computer imagery was set for a mild condition as consisting of a percentage of total lung parenchyma surface area affected no greater than 30%, an average condition of between 30% and 70% and a severe condition as greater than 70% of the lung parenchyma surface area affected.

### 2.4. Research Methods

Initial clinical assessment of the severity of the patient’s condition was conducted using the result of the patient’s physical examination noted in the medical documentation and the type of oxygen therapy implemented. At a later stage, laboratory test results were analysed, including such parameters as the following: D-Dimer, C-reactive protein (CRP), Procalcitonin (PCT), Troponin T (Trop T), Lactate Level (LAC), Fibrinogen, B-Type Natriuretic Peptide (BNP), Interleukin 6 (IL6) and Lactate Dehydrogenase (LDH). Additionally, according to the research protocol, the tumour necrosis factor (TNF) was assessed, as well as the transforming growth factor beta (TNFB1) and arterial blood gas parameters such as Saturation (SpO_2_), arterial oxygen tension (pO_2_), arterial carbon dioxide tension (pCO_2_), alkaline deficiency/excess (BE), bicarbonate level (HCO_3_) and pH. The laboratory results used for analysis were selected on the basis of a review of the literature and a clinical assessment conducted earlier on a group of patients with severe respiratory failure not suffering from COVID-19. The laboratory tests were conducted using arterial blood and the SARSTEDT S-MONOVETTE closed aspiration–vacuum system (Sarstedt, Germany), and analysed in COBAS PRO INTEGRATED SOLUTIONS MODEL ISE/c503/e801, SYSMEX XN-1000, XN-500, BCS XP, ROW COVID-19 (Rotkreuz, Switzerland) Vivalytic laboratory analysers, while arterial blood for blood gas measurements was analysed directly on the ward using a Siemens Rapidpoint 500e Blood Gas Analyser (Münich, Germany). The radiological tests (CT) were described by clinical radiologists so that the assessment was unequivocal and did not raise doubts as to its correctness. Table 1 presents a description of the study group.

### 2.5. Research Documentation Inclusion and Exclusion Criteria

Documentation included in the research was of female and male patients with confirmed COVID-19 requiring oxygen therapy in the form of HFNO, NIV or invasive ventilation. All patients had a physical examination upon admittance with confirmation in the clinical documentation of the severity of the disease, and had a laboratory diagnosis and CT of the chest, and obtained a description with a confirmed entry as to the area of the lungs affected by the disease.

The research exclusion criterium regarded the documentation of patients not requiring oxygen therapy or ventilation as described above and patients who upon admittance had a sudden cardiac arrest irrespective of the success or failure of cardiopulmonary resuscitation. Documentation was also rejected for patients for whom on the basis of the physical examination no information was obtained on the qualification of the patient for an appropriate group of disease severity. Patients were also excluded who due to death or other causes did not undergo CT.

### 2.6. Statistical Data

To conduct the statistical analysis, patient data were entered into an MS Office 2019 spreadsheet. The data were entered and coded in such a way as to make identification of sensitive patient data impossible. The data were stored in an encrypted spreadsheet so as to prevent access by people not involved in the research process. At the stage of preparing the statistical material, the documentation of ten patients was rejected. In eight patients, the CT did not precisely indicate the lung area affected by the disease, and in two patients directly after the first tests were conducted, it became necessary to change the form of oxygen therapy from an NIV mask to invasive ventilation. In order that the above cases did not become disturbance factors, they were partly excluded from the statistical analysis and from comparisons of clinical condition using CT images.

### 2.7. Statistical Analysis

The adopted level of significance was *p* = 0.05, according to which results with *p* < 0.05 represent significant dependencies between the variables. Variables expressed on an ordinal or nominal level were analysed using tests based on the chi-squared distribution. In the case of 2 × 2 tables, continuity correction was used, whereas if the conditions of using the chi-squared test were not fulfilled, the precise Fisher test was applied with extension for tables larger than 2 × 2. Parametric tests (ANOVA variance analysis) or their non-parametric equivalents (the Kruskal–Wallis test) were used to analyse the quantitative variables divided into groups. The selection of the tests was conducted on the basis of the distribution of the variables, which was verified using the Shapiro–Wilk test. Verification of relationships between the variables was conducted using polynomial logistic regression analysis. Calculations were carried out in the R statistical environment version 3.6.0, PSPP software (https://www.gnu.org/software/pspp/) and MS Office 2019.

## 3. Results

The data in Table 1 present the description of the study group by gender and stage of the disease.

The data in Table 2 present a comparison between the clinical assessment and CT image of the severity of a patient’s condition. Patients in a mild clinical condition most often had a mild degree of lung damage on the CT image (45.7%), similarly to patients in an average clinical condition (45.2%). Patients in a severe clinical condition most often had an average degree of damage on the CT image. No statistically significant differences were shown (*p* > 0.05), which indicates that at the initial stage of examining a patient, the clinical assessment is comparative to the assessment obtained using CT imaging.

Table 3 presents the results obtained from laboratory tests in relation to the CT image of lung damage. The data show that upon admittance, BNP, BE and HCO_3_^−^ values, selected due to the degree of lung damage in the CT, differ from one another statistically significantly (*p* < 0.05). For the remainder, the observed differences were not statistically significant. The research demonstrated that along with an increase in the severity of damage to the lungs, there was a statistically insignificant increase in the values of IL6 and LDH, TNF, TGFb1, D-dimer and LAC. People with mild lung damage had minimally higher values of fibrinogen, pH and SpO_2_. People with average damage had minimally higher CRP and pCO_2_, while people with severe damage had higher PCT.

In order to precisely determine between which groups the differences are statistically significant, a post hoc Bonferroni test was conducted, as well as Tukey’s post hoc test—pairwise comparisons. The results of the obtained comparisons are presented in Table 4.

Tabel 4 presents pairwise comparisons of laboratory diagnostic parameters obtained upon admittance of the patient to hospital. Statistically significant differences were obtained in every range for type B natriuretic peptide (BNP). It can be seen that the value of this parameter in the case of average lung damage visible on the CT image was statistically significantly greater than in the case of mild damage (*p* < 0.05). Similarly, in the case of severe lung damage, the BNP values were statistically significantly higher than in the case of average damage (*p* < 0.05). Analogically, in the case of comparison between mild damage and severe damage, the BNP values were higher in the case of severe lung damage. In terms of the level of type B natriuretic peptide, it can be seen that an increase in lung damage, and thus more severe COVID-19, is accompanied by a statistically significant rise in BNP values. A BE alkaline deficiency in comparison to the severity of the disease described in the CT increased between the average and severe condition (*p* < 0.05). It can therefore be seen that in the case of severe COVID-19, BE increases statistically significantly in relation to an average condition. Similarly, in the case of the HCO_3_^−^ parameter, statistically significant differences were noted between people with average lung damage and people with severe damage. People with severe lung damage had a statistically significant (*p* < 0.05) higher HCO_3_ value than people with average damage.

## 4. Discussion

Diagnostic difficulties related to identifying the presence of COVID-19 have existed since the beginning of the pandemic. Research is underway to facilitate the issuing of a correct diagnosis, as well as to determine a patient’s prognosis and correctly assess their condition in a short time. Such an approach is vital from the point of view of early assessment of a patient’s condition upon admittance to hospital, as well as quick identification of the degree of advancement of the disease [10,11,12,13]. In many cases, researchers are seeking solutions to this problem by creating new research models based on the assessment of highly varied factors. The basic elements described in the literature are laboratory diagnostics and imaging, demographic factors and the presence of co-existing illnesses [14,15,16]. Unfortunately, in the current reality, physical examination of patients, on the basis of which clinical conclusions can be drawn about the course of the disease, are replaced with ICT solutions or distance testing and assessment that are often used to give a final diagnosis or to qualify a patient for the appropriate treatment. In their work, Maćkowiak and Gelfman [17,18] clearly draw attention to the fact that technological development and the epidemiological situation should not lead to such a situation in which physical examinations are treated as of secondary importance. Unfortunately, during their literature review, the authors noted difficulties in finding scientific publications indicating the usefulness of a physical examination in assessing and categorising COVID-19 patients. In our research, we made a pioneering attempt to assign patients to the appropriate disease severity group on the basis of a physical examination and the necessary oxygen therapy or ventilation, without the need to conduct imaging diagnostics. The subject of oxygen therapy and ventilation for COVID-19 patients has been broadly described in the literature. In his work, Nair [19] compared the effectiveness of oxygen therapy via NIV and HFNO in relation to the prognosis for patients with severe COVID-19, concluding that he did not obtain an obvious answer as to the advantage of either method. In their work, Menzella et al. [20] proved that the use of non-invasive ventilation decreased the necessity to move patients to the intensive care ward, while Antonelli et al. [21] confirmed that non-invasive ventilation is effective in COVID-19 patients. None of the authors mentioned above made an attempt in their research to use the applied oxygen therapy as an indicator of the severity of COVID-19. In our research, we have shown that examining the patient and qualifying them for the appropriate oxygen therapy can be used as a parameter to indicate the severity of the disease, and as an estimation of the amount of lung area affected by the illness.

In the next stage, the result of the physical examination and the ventilation method used were matched to the laboratory diagnostics parameters. The result obtained clearly indicates differences in the levels of BNP, BE and HCO_3_^−^ depending on the severity of the disease and the amount of lung parenchyma affected by the disease on the CT image. Similar research was presented by Ghelfi AM [22], in which an increase was demonstrated in NT pro-BNP values proportionally to the severity of the condition of the COVID-19 patient. In the same study, the level of troponin T was assessed, which similarly to our study showed no statistically significant differences. Salvatore C et al. [23] compared laboratory results with the severity of lung damage on the CT image. This study was conducted on patients divided into three groups depending on the severity of the disease. On the basis of their results, they showed that CRP, leukocytes, neutrophils, LDH, D-Dimer and troponin values were statistically significantly higher in patients with a more severe condition. In their research, Zhang et al. [24] demonstrated that the level of some inflammatory markers increased along with the severity of the disease. They proved that the level of CRP and PCT increased in severely ill patients, which in our research was not confirmed statistically. Analysing our results in terms of laboratory norms, it can be seen that some parameters increased depending on the severity of the disease and the amount of lung parenchyma affected by the disease, but that these values were not statistically significant for the research model. Considering the results from the perspective of arterial blood gas analysis, it was possible to identify two key elements that indicate the severity of the disease and the degree of lung infection. In the analysed material, a statistically significant increase was observed in BE and HCO_3_^−^. In their research, Turcato et al. [25] proved there was a rise in pCO_2_ and a drop in pO_2_ in patients admitted to hospital with severe COVID-19. They correlated these results with CT images and demonstrated statistically significant dependencies. However, our research did not reveal a correlation between changes in pO_2_ and pCO2 depending on the severity of the disease. Sanghani et al. [26] showed the presence of respiratory alkalosis and metabolic alkalosis secondary to it. Our research partly confirmed these results and indicated an increase in BE and HCO_3_^−^ parameters, but no significant changes were observed in terms of pH. A careful review of the literature shows that the laboratory markers used in our research model are often used for COVID-19 diagnostics. Physical examination and oxygen therapy are not commonly used to determine the predictive model or to assess the severity of the disease. It must be underlined that previously, nobody had taken the effort to combine these factors and use them as a diagnostic tool. The model developed in this research can be an effective tool for assisting in determining the severity of COVID-19 and the degree to which the lung parenchyma is affected by the disease.

## 5. Limitations

The main limitation of the research is that it was conducted in only one centre, while to obtained more transparent results, it would be necessary to repeat the research in multiple centres. A limitation is the lack of opportunity to implement the research project in test conditions, caused by the research being conducted in difficult epidemiological conditions. The research model also requires further work so as to avoid loss of data and an amount of research material while the research is in progress.

## 6. Conclusions

1. On the basis of a physical examination and the applied oxygen therapy, a patient can be assigned to the appropriate group of severity of COVID-19.
2. On the basis of BNP, HCO_3_ and BE parameters obtained from arterial blood, a patient can be assigned to the appropriate group of severity of COVID-19.

## Figures and Tables

**Table 1:** Description of study group by gender and stage of the disease.

**Gender**	**[*n*]**	**[%]**
Female	46	48.40%
Male	49	51.60%
**Stage of disease on the basis of CT scan**	**[*n*]**	**[%]**
Mild condition (<30% of lung parenchyma affected)	34	39.10%
Average condition (30–70%)	35	40.20%
Severe condition (>70%)	18	20.70%
**Stage of disease on the basis of clinical condition**	**[*n*]**	**[%]**
Mild condition (HFNO)	39	41.90%
Average condition (NIV ventilation)	35	37.60%
Severe condition (invasive ventilation)	19	20.40%

**Table 2:** Differences in clinical assessments of disease severity with reference to first computed tomography result.

			Clinical Assessment of Stage of Disease			Test Result
			Mild Condition	Average Condition	Severe Condition	
Computed tomography result	Mild condition	N	16	14	3	*χ*^2^ = 6.352 *df* = 4 *p* = 0.174
		%	45.7%	45.2%	15.8%	
	Average condition	N	14	10	10	
		%	40.0%	32.3%	52.6%	
	Severe condition	N	5	7	6	
		%	14.3%	22.6%	31.6%	
Total		N	35	31	19	
		%	100.0%	100.0%	100.0%	

*χ*^2^—test statistic; *df*—degrees of freedom; N—number; and *p*—statistical significance.

**Table 3:** Laboratory test results obtained upon patient admittance to hospital in comparison to assessment of disease severity in the first computed tomography imaging.

Parameter	CT Result	*χ* ^2^	*df*	*p*	*Min*	*Max*	*Me*
BNP [pg/mL]	Mild condition	37.18	2	<0.001	0.71	15.05	3.28
	Average condition				6.29	20.47	10.73
	Severe condition				1.38	24.39	15.66
IL6 [pg/mL]	Mild condition	12.06	2	0.055	0.03	41.60	4.07
	Average condition				0.21	534.40	10.58
	Severe condition				2.68	471.60	17.02
LDH [U/L]	Mild condition	17.27	2	0.055	5.90	26.99	11.88
	Average condition				7.74	119.80	13.59
	Severe condition				10.35	38.91	19.80
TNF [pg/mL]	Mild condition	3.19	2	0.202	27.51	6691.00	45.40
	Average condition				33.41	5219.00	49.11
	Severe condition				20.73	769.00	57.44
TGFb1 [pg/mL]	Mild condition	5.30	2	0.071	35.67	430.80	47.08
	Average condition				39.97	681.10	53.26
	Severe condition				44.06	651.90	55.01
D-dimer [ng/mL]	Mild condition	4.58	2	0.101	264.90	31,382.59	874.58
	Average condition				346.89	36,218.18	969.80
	Severe condition				522.50	14,556.26	1830.43
CRP [mg/L]	Mild condition	2.88	2	0.237	15.70	278.00	81.40
	Average condition				4.90	327.00	103.00
	Severe condition				4.90	290.00	89.60
PCT [ng/mL]	Mild condition	0.39	2	0.823	0.02	1.78	0.13
	Average condition				0.02	1.31	0.12
	Severe condition				0.04	0.64	0.15
Trop T [ng/mL]	Mild condition	1.50	2	0.473	0.00	0.05	0.01
	Average condition				0.00	0.09	0.01
	Severe condition				0.00	0.53	0.01
LAC [mg/dL]	Mild condition	0.97	2	0.617	0.76	3.45	1.47
	Average condition				0.85	4.02	1.56
	Severe condition				0.98	3.27	1.69
Fibrinogen [g/L]	Mild condition	0.91	2	0.635	1.09	8.91	7.02
	Average condition				2.35	8.91	6.11
	Severe condition				3.40	8.91	6.79
pH	Mild condition	2.44	2	0.295	7.31	7.56	7.47
	Average condition				7.31	7.56	7.45
	Severe condition				7.36	7.54	7.46
SpO_2_ [%]	Mild condition	2.32	2	0.313	53.50	98.50	91.40
	Average condition				46.80	98.90	87.00
	Severe condition				39.50	99.40	90.65
pO_2_ [mmHg]	Mild condition	1.73	2	0.421	42.70	138.10	74.30
	Average condition				25.20	155.40	72.50
	Severe condition				19.00	113.20	78.05
BE [mmol/L]	Mild condition	6.30	2	0.043	−5.50	8.00	1.75
	Average condition				−5.30	6.60	1.20
	Severe condition				−2.10	8.00	1.85
pCO_2_ [mmHg]	Mild condition	0.27	2	83	0.766	37.78	6.24
	Average condition					38.83	7.52
	Severe condition					39.13	5.38
HCO_3_ [mmol/L]	Mild condition	5.26	2	83	0.007	24.62	3.95
	Average condition					22.93	4.07
	Severe condition					26.43	3.24

*χ*^2^—test statistic; *df*—degrees of freedom; *p*—statistical significance; *Min*—minimum result; *Max*—maximum result; and *Me*—median.

**Table 4:** Pairwise comparison of statistically significant laboratory diagnostic results in relation to lung damage on computed tomography imaging.

	Computed Tomography Result		*p*	
BNP [pg/mL] ^1^	mild	average	<0.001	***
	mild	severe	<0.001	***
	average	severe	0.014	*
BE [mmol/L] ^1^	mild	average	0.129	
	mild	severe	1.000	
	average	severe	0.049	*
HCO_3_ [mmol/L] ^2^	mild	average	0.118	
	mild	severe	0.319	
	average	severe	0.006	**

^1^ Bonferroni post hoc test; ^2^ Tukey’s post hoc test; * *p* < 0.05; ** *p* < 0.01; *** *p* < 0.001; and *p*—statistical significance.

## Data Availability

The data presented in this study are available on request from the corresponding author.
